# Lifespan changes in postural control

**DOI:** 10.1038/s41598-022-26934-0

**Published:** 2023-01-11

**Authors:** Nathan Van Humbeeck, Reinhold Kliegl, Ralf T. Krampe

**Affiliations:** 1 Brain and Cognition Group, University of Leuven (KU Leuven), Leuven, Belgium; 2 Department of Sports and Health Sciences, University of Potsdam, Potsdam, Germany

**Keywords:** Paediatric research, Geriatrics

## Abstract

Lifespan development of postural control shows as an inverted U-shaped function with optimal performance in young adults and similar levels of underperformance in children and older adults. However, similarities in children and older adults might conceal differences in underlying control processes. We mapped out age-related differences in postural control using center-of-pressure trajectories of 299 participants ranging from 7 to 81 years old in three tasks: stable stance, compromised vision, and narrowed base of support. Summary statistics (path length, ellipse area) replicated the well-known U-shape function also showing that compromising vision and narrowing the base of support affected older adults more than children. Stabilogram diffusion analysis (SDA) allows to assess postural control performance in terms of diffusion at short (< 1 s) and longer timescales. SDA parameters showed the strongest short-term drift in older adults, especially under compromised vision or narrowed base of support conditions. However, older adults accommodated their poor short-term control by corrective adjustments as reflected in long-term diffusion under eyes closed conditions and initiating anti-persistent behavior earlier compared with children and young adults in tandem stance. We argue that these results highlight the adaptability of the postural control system and warrant a reinterpretation of previous postural control frameworks.

## Introduction

Achieving and maintaining a stable upright stance is an accomplishment relevant to individuals of all age groups. Challenges and risks inherent to this accomplishment, however, differ between age groups. Instability and falling are necessary aspects of motor development in early childhood and they play an essential role in adolescents and young adults practicing sports like gymnastics, martial arts, or skateboarding. In later adulthood instability is associated with fear and falling poses a risk of traumatic or even life-threatening injuries^1,2^. The act of acquiring a stable state of balance is referred to as postural control^3^. Research into the development of postural control has largely proceeded in independent contexts. Using young adults as a reference group it either covers age-ranges from birth to adolescence or it focusses on later decades of life and risk of falling. Experimental studies including both ends of the lifespan are the exception^4^. Merging different pieces of evidence, the developmental trajectory of postural stability appears as a U-shaped function with lowest levels of performance at both ends of the lifespan, much like for most cognitive and sensorimotor abilities. However, these similarities in underperformances of children and older adults may conceal differences in underlying mechanisms. So far, a comprehensive approach integrating related changes is missing.

In this study, we propose a framework that focuses on age-related changes for control processes operating at short or long timescales and their temporal dynamics. We apply traditional summary statistics as well as stabilogram diffusion analysis to center-of-pressure (CoP) trajectories from 299 individuals aged between 7 and 81 years. Participants performed three postural control tasks designed to differentially challenge proprioceptive and visual information processing. By determining parameters acting at short and long timescales, we account for changes in postural stability as they emerge from a lifespan perspective.

An important characteristic of postural control is its inherent instability. Even in healthy adults, stable upright stance is subject to oscillatory sway, the control of which requires cooperating mechanisms presumably operating at different timescales^5^. An early hypothesis by Winter et al. proposed mechanical stiffness of the musculoskeletal system as the only component needed to stabilize the body during unperturbed bipedal stance^6^. This would minimize operating demands on the central nervous system while providing instantaneous responses to diverging motion. Though damping and stiffness due to passive and active structures are considered essential in control processes operating at short timescales, many studies have shown that time-delayed feedback mechanisms are also required in order to stabilize the body, especially over longer periods of time^7^. In order to efficiently execute these time-delayed corrective feedback movements, sensory input from proprioceptive, visual, and vestibular systems has to be gathered and reweighted. This multisensory integration facilitates positional awareness and motion perception, allowing us to adjust our bodily movement with high precision. The efficiency of these control mechanisms can be determined by assessing postural sway using measures of CoP. The CoP is defined as the point at which the pressure of the body would be if it were concentrated in one spot^8^. Quantification of CoP movements traditionally involves summary statistics such as the total CoP displacement (path length) or the area covered by CoP movements. These measures suffer from important limitations, however. CoP area provides an appropriate summary measure of long-term position-based behavior, but it does not capture short-term balance dynamics. Participants are evaluated based on the range of CoP movement regardless of the quality of moment-to-moment postural control^9,10^. In addition, the validity of the CoP area heavily relies upon the assumption of stationarity, implying an objective reference point around which CoP movements occur. Small weight shifts can drastically inflate the area measure despite excellent overall performance. In contrast, displacement measures describe short-term, non-stationary CoP movements without taking its long-term position into account. These measures follow the common assumption that the postural control system is tuned to minimize sway at any moment in time. This idea has been disputed by several authors arguing that the balance system adopts a strategy of stabilization rather than sway minimization^11^. Recently, theorists have recognized the exploratory character of CoP movements emphasizing that increased displacement at short timescales provides proprioceptive information enhancing long-term positional control and awareness^12^.

The described shortcomings of traditional summary statistics highlight a key distinction in extant models of postural control, namely the one between short and long timescale control mechanisms. However, the composition of short and long timescale control processes as well as the dynamics underlying their transition remain a matter of debate. Interactions between short- and long-term mechanisms in postural control call for data analyses techniques reflecting non-stationary behavior and taking long-term position-based control into account. From a developmental perspective the questions arise whether different mechanisms mature or decline at similar rates and when their interaction is optimized during lifespan development.

Several studies have depicted the trajectory of postural control development in children, adolescents and young adults using summary statistics like CoP path length or CoP area^13–15^. Likewise, numerous studies found age-related increases in these measures for comparisons of young and older adults^16–19^. Most authors agree that optimal postural control is reached in young adulthood, remains relatively stable in healthy adults until the early 60s and deteriorates at accelerating rates after the 7th decade of life^16,20,21^. Several studies attempted to link age-related differences to certain mechanisms. However, their focus was typically on a limited age-range and single control mechanisms.

While the developmental milestone of upright stance is typically reached around the end of the first year of life, mature multisensory integration strategies are not attained before young adulthood^22,23^. Presumably, the continuous need to recalibrate the proprioceptive system to body growth determines differences in sensory integration and reweighting strategies. Shumway-Cook and Woollacott^24^ observed a dominant visual control strategy in toddlerhood and early childhood which only began to shift towards adult-like multisensory strategies around ages 4–6. In addition, the authors found superior balance performance in 7–10 year-old children through improved inhibition of conflicting sensory modalities. More recently, Sinno et al.^22^ provided further evidence for a primarily visuo-vestibular dependence in children. The authors argued that efficient reweighting of sensory modalities continued improving into early adulthood.

Adult age-related deterioration of the postural control system results from several factors. Changes in muscle spindle morphology and synaptic transmission reduce the reliability of the proprioceptive system^25^. Apart from specific alterations in sensory systems, global changes in the central nervous system like declining grey and white matter integrity hamper the efficiency of multisensory integration and reduce the processing speed required to generate accurate motor responses^25,26^. Using an experimentally moving room, Wade et al.^17^ found that older adults rely more on their visual system in order to maintain postural control compared with young adults.

While evidence is not unequivocal^27^, most studies agree that disturbances of visual input have more detrimental effects on balance in older compared with young adults^28–31^. Another specific characteristic of balance in older adults is muscle-coactivation. Nagai et al.^18^ found higher muscle co-activation during static standing in older compared with young adults. In older but not young adults, CoP area was significantly correlated with muscle co-activation in line with the notion that higher coactivation signals higher challenge^32^, either because of the individual's poor balance control or the type of situation and task.

Collins and De Luca^33^ introduced a pioneering method focusing the notion of control processes operating at different timescales, stabilogram diffusion analysis (SDA). Based on Einstein’s theory of Brownian motion, the SDA analyzes mean squared CoP displacement at different timescales (in contrast with traditional approaches in which time is analyzed as a continuous variable). Collins and De Luca argued that SDA plots reveal two distinct regions which reflect the workings of postural control mechanisms on short and long timescales, respectively. The first region (short timescales) is characterized by persistent displacement which implies, on average, continuous motion in a certain direction (persistence). The second region denotes longer timescales characterized by more stationary anti-persistent behavior. The transition point separating those timescales is called the critical point. Collins and De Luca fitted linear regression lines to derive short-term and long-term diffusion coefficients. According to their interpretation, behavior at short timescales reflects open-loop control schemes tempering the inherently unstable body. That is, descending motor commands activate stabilizing muscles which provides a continuous resistance to movement. Consecutively, once a certain threshold is reached, long-term closed-loop mechanisms come into effect resulting in anti-persistent corrective feedback motion.

In later studies, Collins and De Luca^34,35^ applied their method to the study of adult-age differences and the effect of visual input on postural control mechanisms operating at short and long timescales. They found that during stable stance, older adults showed steeper short-term but shallower long-term slopes than young adults. In addition, older adults showed longer critical time intervals. The authors suggested that steeper short-term slopes in older adults may reflect a postural control strategy in which they increase lower limb muscle co-activation. According to the authors older adults simultaneously compensated for this decrease in short-term stability and longer processing delays by improving long-term correction. Collins and De Luca assumed that the increase in critical time intervals reflected age-related delays in activating long-term error correction mechanisms. Regarding the effect of visual input on short and long timescale mechanisms, they hypothesized that long-term control (reflecting multisensory integration and corrective adjustments) was delayed due to the removal of visual information. Surprisingly, their results showed that this was only true for half of their participants, while the other half actually improved long-term control under compromised vision.

Authors applying SDA modelling typically adopted the open- vs closed-loop view proposed in the original work^33^, though neurophysiological underpinnings and component processes related to this two-part behavior remain an issue of debate. Peterka^36^ modelled a closed-loop neural controller in an inverted pendulum model and translated SDA parameters to neurophysiological component processes of postural control like stiffness, damping, and time delay. Using simulation, he showed that a two-part function does not necessarily require the assumption of two different control systems one of which only works with feedback delay. In his model, the characteristic bipartite shape arose from the combined effects of different factors including the stiffness, damping and inertia of the musculoskeletal system as well as the feedback time-delay.

In general, discrepancies and uncertainties in terms of the interpretation of stochastic modelling of the CoP have limited its potential which might explain why traditional summary statistics such as CoP displacement are still considered the gold standard in CoP quantification. The aims of our study are to improve the interpretation of stochastic modeling parameters, particularly when different stability contexts are considered, and to determine how component processes of postural control and their interaction change across the lifespan.

Our review of age-comparative studies suggests a U-shape development of postural stability across the lifespan. While existing studies had considerable value delineating developmental trajectories of postural control, they remained silent regarding underlying mechanisms. We argue that three reasons have so far limited progress in the domain. First, age-comparative studies focused on a small age-range typically comparing either children to young adults or young and older adults. Second, experimental manipulations were limited to targeting but one underlying candidate mechanism. Third, previous studies for the most part used summary statistics like CoP path length or CoP area which reflect the joint contributions of component mechanisms without determining their efficiencies and differential contributions.

To overcome these limitations, we investigated postural control in a large-scale sample of 299 participants between ages 7–81 years. We compared their performances in three tasks assessing stability under stable stance, compromised vision (i.e., eyes closed), and narrowed base of support (i.e., tandem stance) conditions.

Our approach is inspired by the discussions related to the interpretation of SDA parameters^36,37^. Here we side with three general lines of arguments. First, we side with theoretical proposals^11,12^ that the postural control system optimizes stabilization rather than minimizing sway. Second, we follow Peterka^36^ in that we doubt that the two regions empirically identified in SDA plots represent pure separations of open- and closed-loop control mechanisms. Finally, we assume that short- and long-timescale control mechanisms as well as dynamics of their transition are at least partly under the individual's control and can thus be adjusted to contextual challenges. These assumptions have important implications for changes in SDA parameters once conditions with smaller stability boundaries, reduced availability of feedback, or groups differing in sensorimotor maturation are considered. From the assumption that vision contributes to balance control in a feedback time-delayed manner, we predict that the removal of visual information will affect long timescale region diffusion. Instability induced by narrowing base-of-support will first and foremost increase short-term diffusion. We predict a performance advantage for young adults in all parameters. Though postural control in both children and older adults is highly reliant on visual information, we predict pronounced effects of the removal of visual information in children. On the other hand, as higher challenge has been associated with muscle co-activation^18,32,38^ and steeper short-term slopes^18^ in older adults, we predict that the narrowed base of support will disproportionately affect short timescale displacement (i.e. increased short-term diffusion).

## Results

**Figure 1:**
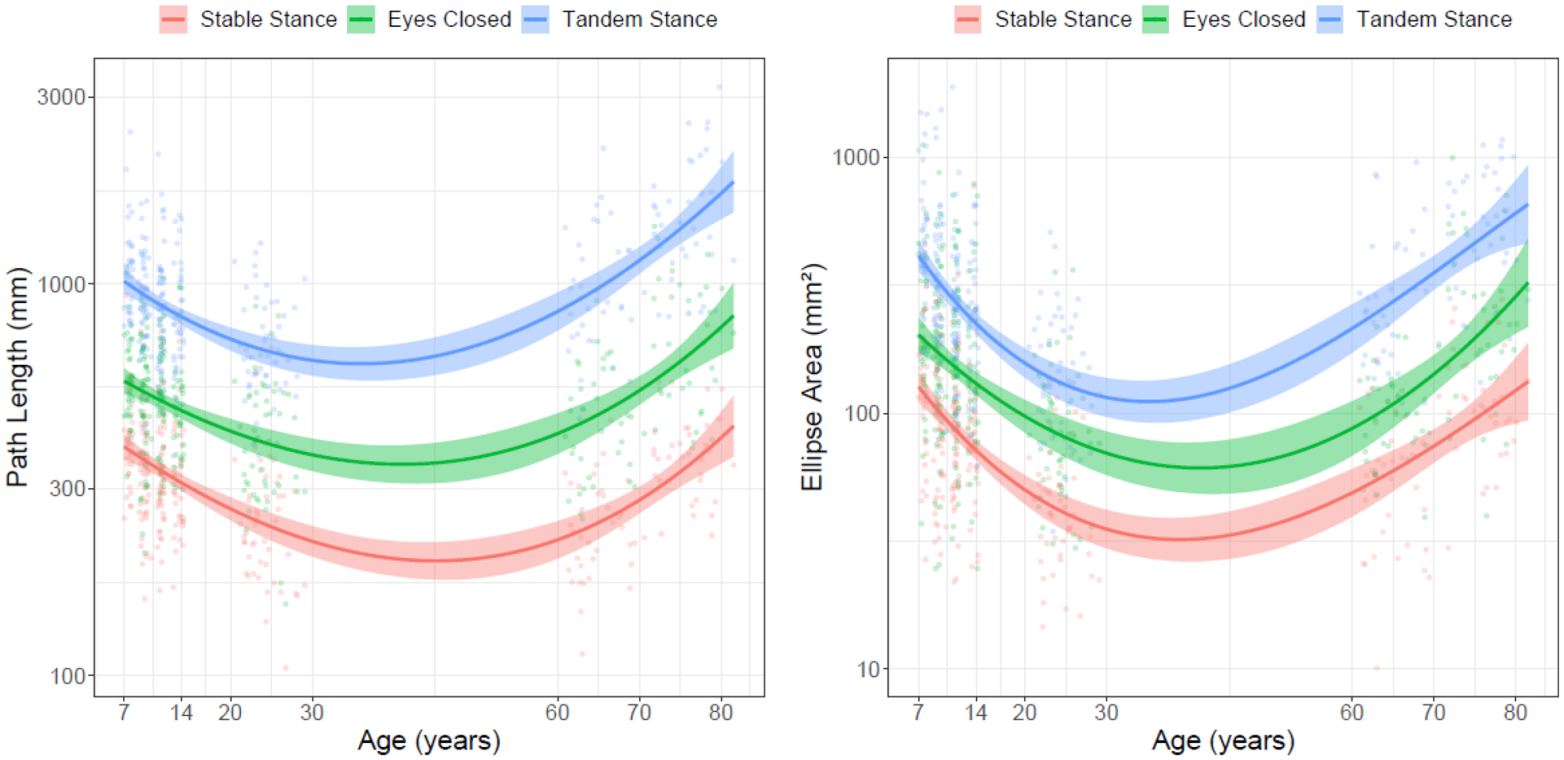
U-shaped pattern of postural stability across the lifespan for planar path length (left) and ellipse area (right) indicating significant differences between experimental conditions as well as reliable age-related changes within children and older adult groups (see Supplementary Table 1 and 2). Error envelope = ± 1SE.

Figure 1 shows that, using traditional summary statistics, our data conform to the U-shaped pattern of postural stability when mapped across the lifespan. The LMM on planar path length (see Supplementary Table 1–12 for statistics of this and all following LMM analyses) indicated less displacement in stable stance when compared with eyes-closed (z = 35.294, *p* < 0.001) and tandem stance (z = 62.898, *p* < 0.001) and this pattern generalized to all age groups (t’s > 15.575, *p* < 0.001, Table 1). Young adults' displacement was smaller compared with children (z = 6.767, *p* < 0.001) as well as older adults (z = 6.872, *p* < 0.001) and this was robust across conditions (t’s > 2.482, *p* ≤ 0.044, Table 1). Condition by age group interactions indicated pronounced effects of removal of visual information (z = 4.373, *p* < 0.001) and narrowing of the base of support (z = 7.279, *p* < 0.001) in older compared with young adults. Nested effects of age showed lower displacement with increasing age in children (z = 3.235, *p* = 0.001) and higher displacement with increasing age in older adults (z = 6.797, *p* < 0.001).

**Table 1:** Postural control measures for three experimental conditions and age groups. Note that these values do not depict the transformed measures used for statistical analysis. M = mean, SD = standard deviation. ^a^ denotes significant (*p* < 0.05) difference when compared with young adults using unpaired t-tests. ^b^ denotes significant (*p* < 0.05) difference in children when compared with older adults using unpaired t-tests. Post-hoc analyses are Bonferroni corrected.

	Children		Young adults		Older adults	
	M	SD	M	SD	M	SD
**Stable stance**						
Planar path length (mm)	361.35^a,b^	125.52	263.59	106.31	305.66^a^	100.82
Ellipse area (mm^2^)	116.71^a^	93.67	48.39	26.70	92.37^a^	57.34
Short-term diffusion coefficient (mm^2^ s^−1^)	11.55^a^	7.75	6.16	3.24	9.70^a^	6.08
Long-term diffusion coefficient (mm^2^ s^−1^)	2.06^a^	2.00	0.91	0.60	1.59^a^	1.32
Critical time interval (s)	0.76^a^	0.29	0.63	0.22	0.82^a^	0.35
Critical mean squared displacement (mm^2^)	15.61^a^	11.39	6.38	2.92	13.82^a^	9.87
**Eyes closed**						
Planar path length (mm)	540.80^a^	166.14	420.56	155.79	602.10^a^	261.28
Ellipse area (mm^2^)	208.98^a^	168.34	99.28	68.95	202.86^a^	182.11
Short-term diffusion coefficient (mm^2^ s^−1^)	27.69^a^	18.39	17.96	14.36	40.52^a^	47.75
Long-term diffusion coefficient (mm^2^ s^−1^)	2.94^a,b^	3.46	1.25	1.09	1.70	2.42
Critical time interval (s)	0.82	0.32	0.89	0.41	0.96	0.46
Critical mean squared displacement (mm^2^)	42.21^a^	31.72	26.54	16.42	62.42^a^	59.17
**Tandem stance**						
Planar path length (mm)	949.05^a,b^	315.06	723.26	211.68	1284.85^a^	558.87
Ellipse area (mm^2^)	368.99^a^	295.28	161.25	93.49	449.43^a^	294.44
Short-term diffusion coefficient (mm^2^ s^−1^)	69.61^a,b^	51.70	38.63	20.69	116.26^a^	100.48
Long-term diffusion coefficient (mm^2^ s^−1^)	3.69^a^	4.60	1.78	1.70	4.43^a^	5.37
Critical time interval (s)	0.49^b^	0.19	0.44	0.18	0.38	0.09
Critical mean squared displacement (mm^2^)	65.22^a^	56.55	30.23	15.11	82.66^a^	71.75

Ellipse area for COP sway (right panel Fig. 1) showed the same differences among conditions: areas were smaller in stable stance compared with eyes-closed (z = 17.467, *p* < 0.001) and tandem stance (z = 31.027, *p* < 0.001) (see Supplementary Table 2). This pattern generalized to all age groups (t’s > 8.442, *p* < 0.001, Table 1). Young adults showed smaller sway areas than children (z = 9.118, *p* < 0.001) and older adults (z = 7.588, *p* < 0.001) and this main effect generalized across conditions (t’s > 4.902, *p* < 0.001, Table 1). A condition by age group interaction showed that compared with young adults, older adults were disproportionately affected by narrowing the base of support (z = 2.991, *p* < 0.004). Nested effects of age revealed smaller ellipse areas with age in children (z = 4.450, *p* < 0.001) and larger ellipse areas with age in older adults (z = 5.862, *p* < 0.001).

Separate LMMs directly contrasting children and older adult groups confirmed the visual impression: neither path length (z = 1.298, *p* = 0.195) nor ellipse area (z = 0.452, *p* = 0.652) showed main effects of age group. Condition by age group interactions for the path length analysis showed pronounced effects of the removal of visual information (z = 7.680, *p* < 0.001) and narrowing of the base of support (z = 11.238, *p* < 0.001) in older adults. The latter effect was replicated in the ellipse area analysis (z = 4.461, *p* < 0.001).

### Stabilogram diffusion parameters

**Figure 2:**
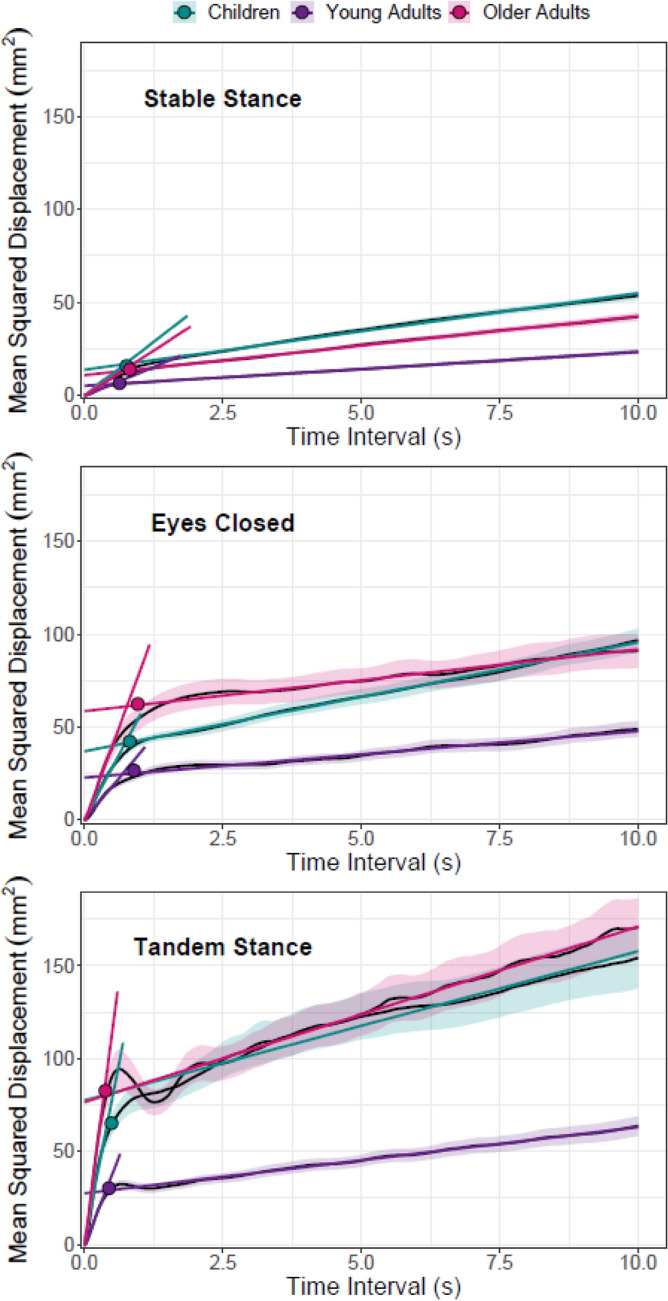
Averaged stabilogram diffusion plots with fit lines based on parameter estimates for short- and long-term diffusion coefficients in stable stance, eyes closed and tandem stance conditions. Symbols denote the critical point. Error envelope = ± 1SE.

Stabilogram diffusion plots (Fig. 2) revealed shallower slopes for short-term regions in stable stance compared with eyes closed (z = 27.687, *p* < 0.001) and tandem stance (z = 49.598, *p* < 0.001) conditions and this was true across age groups (t’s > 13.138, *p* < 0.001, Table 1). Young adults performed better (smaller short-term diffusion coefficients) when compared with children (z = 6.488, *p* < 0.001) and older adults (z = 7.197, *p* < 0.001), a main effect generalizing across conditions (t’s > 3.885, *p* < 0.001, Table 1). A condition by age group interaction revealed that removal of visual information (z = 2.240, *p* = 0.026) and narrowing the base of support (z = 4.939, *p* < 0.001) had a pronounced effect on older compared with young adults. Long-term regions showed shallower slopes in stable stance when compared with tandem stance (z = 6.875, *p* < 0.001) and this was true in all age groups (t’s > 3.190, *p* ≤ 0.007, Table 1). Young adults showed shallower long-term slopes than children (z = 6.057, *p* < 0.001) and older adults (z = 3.352, *p* < 0.001). While this main effect was robust across conditions when children were compared with young adults (t’s > 3.802, *p* < 0.001, Table 1), the effect was only robust in stable and tandem stance (t’s > 3.742, *p* < 0.001,Table 1) when comparing young to older adults. Moreover, a crossover interaction indicated that the effect of removing visual information was less pronounced in older adults when compared with young adults (z = 2.113, *p* = 0.035).

The critical time interval in stable stance was shorter than in the eyes closed condition (z = 5.424, *p* < 0.001). At the same time, the time interval in tandem stance was shorter than in stable stance (z = 16.146, *p* < 0.001). These trends generalized to all age groups (t’s > 6.436, *p* < 0.001, Table 1) except from the shorter time intervals found in stable stance relative to eyes closed which was robust only in young adults (t = 4.553, *p* < 0.001). A condition by age group interaction indicated that children had pronounced increases in critical time interval compared with young adults when deprived of visual information (z = 2.756, *p* = 0.006). On the other hand, older adults showed pronounced reductions in the critical time interval compared with young adults when base of support was narrowed (z = 3.904, *p* < 0.001).

Critical displacement showed reliable effects of task condition: lower critical displacement was found in stable stance when compared with eyes closed (z = 37.516, *p* < 0.001) and tandem stance (z = 43.776, *p* < 0.001). These effects generalized to all age groups (t’s > 17.981, *p* < 0.001, Table 1). Young adults showed significantly lower critical displacement when compared with children (z = 8.196, *p* < 0.001) and older adults (z = 8.368, *p* < 0.001) and this effect was robust across experimental conditions (t’s > 4.438, *p* < 0.001, Table 1). A crossover interaction indicated that the effect of removing visual information was less pronounced in children when compared with young adults (z = 4.680, *p* < 0.001). Further, a condition by group interaction revealed pronounced effects of narrowing the base of support in older when compared with young adults (z = 1.987, *p* < 0.048).

**Figure 3:**
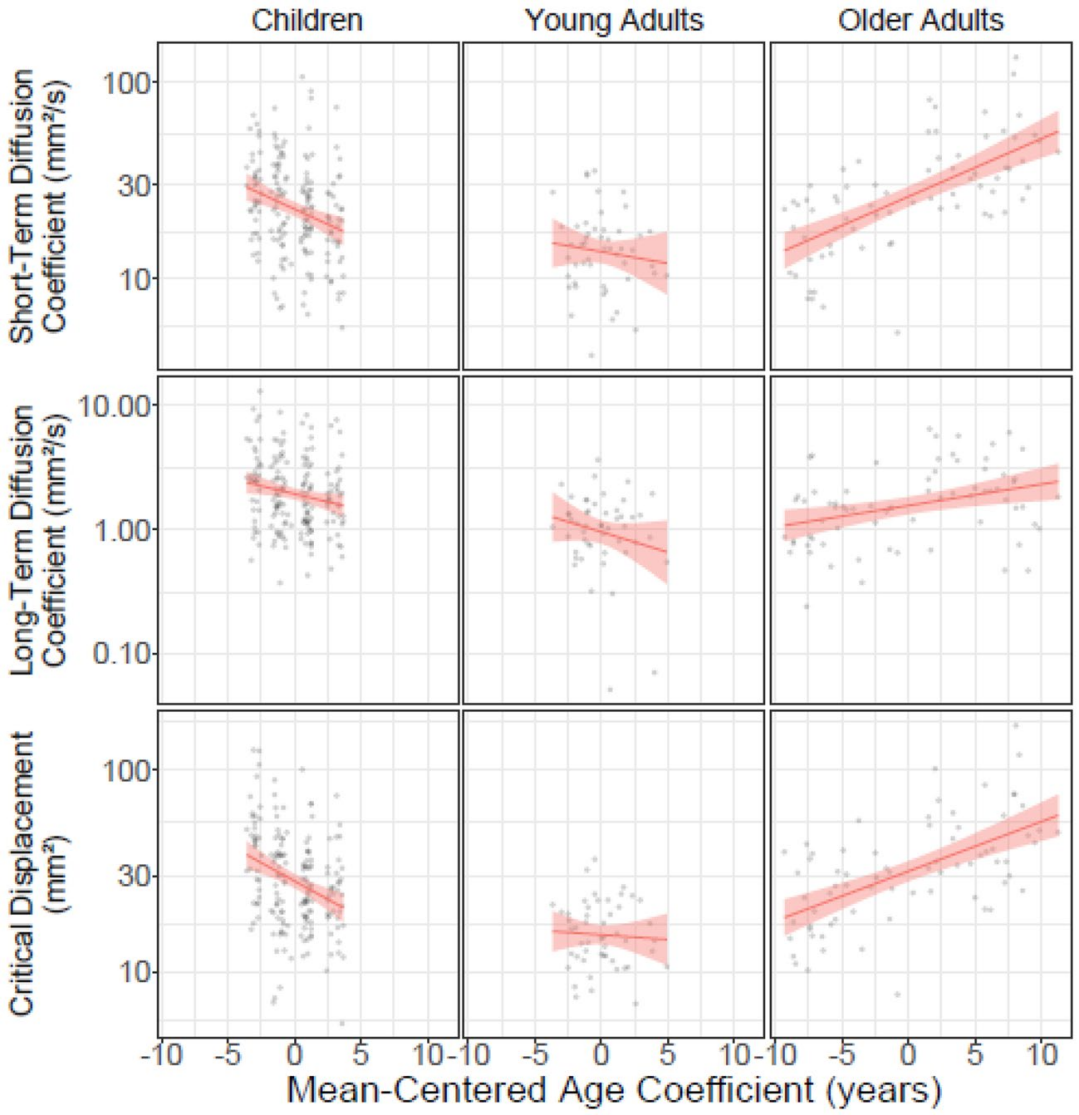
Nested age effects for the short-term diffusion coefficient, long-term diffusion coefficient and critical displacement. Error envelope = ± 1SE.

Nested age effects (see Fig. 3) reflected children's developmental gains in terms of reduced short-term (z = 4.146, *p* < 0.001) and long-term diffusion (z = 2.916, *p* = 0.004). Reliable age-related decline (i.e., increases) was observed in short (z = 7.187, *p* < 0.001) and long-term diffusion (z = 2.903, *p* = 0.004) coefficients in older adults. Nested effects did not contribute to the critical time interval. However, for critical displacement, age-related decreases in displacement were robust in children (z = 4.948, *p* < 0.001), as were age-related increases in displacement in older adults (z = 6.191, *p* < 0.001).

### Comparison children and older adults

The additional LMMs directly contrasting children and older adults yielded the same findings with respect to experimental conditions as the previous analysis. For short-term diffusion coefficient, a condition by group interaction revealed pronounced effects in older adults when deprived of visual information (z = 4.544, *p* < 0.001) or when the base of support was narrowed (z = 7.065, *p* < 0.001). Further, children had steeper long-term slopes compared with older adults (z = 2.517, *p* = 0.012), which was robust only in the eyes closed condition (t = 3.735, *p* < 0.001). A condition by age group interaction revealed that removal of visual information had a pronounced effect on children when compared with older adults (z = 2.517, *p* = 0.012). For the critical time interval we found a condition by group interaction indicating a disproportionate decrease in older adults when the base of support was narrowed (z = 4.176, *p* < 0.001). Lastly, the critical displacement also showed condition by group interactions indicating pronounced increases in older adults when visual information was removed (z = 5.965, *p* < 0.001) or the base of support was narrowed (z = 4.346, *p* < 0.001).

## Discussion

We analyzed lifespan development of postural control mechanisms using traditional summary statistics and stabilogram diffusion analysis. Our working hypothesis was that similar gross levels of postural stability at both ends of the lifespan reflected in traditional summary statistics concealed differences in underlying mechanisms. Our results for planar path length and COP-ellipse area showed a U-shaped developmental trajectory with younger adults outperforming children and older adults. This was corroborated by age-related gains within children- and age-related declines within older adult-groups. Our experimental manipulations to visual input and base of support were effective in all groups leading to proportional increases in children compared with young adults and pronounced increases in older adults. While children and older adults did not differ at the level of main effects for summary statistics, removal of visual input and reduction of base of support affected older adults more than children.

A more complex picture emerged from the SDA parameters. Both compromising vision as well as narrowing base of support reliably increased short-term diffusion across groups with pronounced effects in older adults. These interactions also emerged in the direct comparison between children and older adults, again highlighting the extra challenge for the latter group. Long-term diffusion was affected by narrowing the base of support in all groups. We found no effect of compromising vision on long-term diffusion in young adults, replicating results from Collins and De Luca^34,39^. Children showed equally poor long-term control in eyes closed as in stable stance conditions. However, relative to children and young adults, older adults managed to improve their long-term control when vision was compromised. Presumably, this reduction in long-term diffusion acts as a compensation for their pronounced short-term displacements.

For critical time intervals, our experimental manipulations showed opposite effects. Compromising vision led to an increase in critical time intervals whereas narrowing the base of support led to a decrease. With regards to age differences, children showed higher critical time intervals than young adults in stable stance but were reliably less affected by the removal of visual information. Our findings for stable stance are in line with earlier studies which found increased critical time intervals in older compared with young adults^35,37^. At the same time, older adults showed pronounced decreases in critical time intervals when the base of support was narrowed.

Our results do not support the original interpretation that increased critical time intervals in older age reflect higher feedback time delays due to age-related slowing. Instead, they point to an adaptive role of critical time intervals accommodating task difficulty and the individual’s capacity to cope with a context. Children’s poor long-term control arguably accelerates initiation of corrective postural adjustments in eyes-closed conditions, while elevated short-term displacement in older adults promotes rapid postural adjustments in tandem stance. This interpretation is in line with Peterka^36^ who also suggested that feedback time delay does not play a dominant role in the determination of the critical point. Further support for our interpretation comes from critical displacement, which showed a similar pattern as summary statistics with respect to experimental manipulations and age-differences in the reaction to them. Children were less affected by the removal of visual information, which could be explained by expedited corrective adjustments. Pronounced short-term increases in displacement in older adults gave rise to accelerated corrective adjustments as well. We believe that in situations of jeopardized stability short-term displacement becomes the dominant factor in initiation of corrective adjustments. Older adults are more affected by challenges than children, but they also have better compensatory mechanisms.

**Figure 4:**
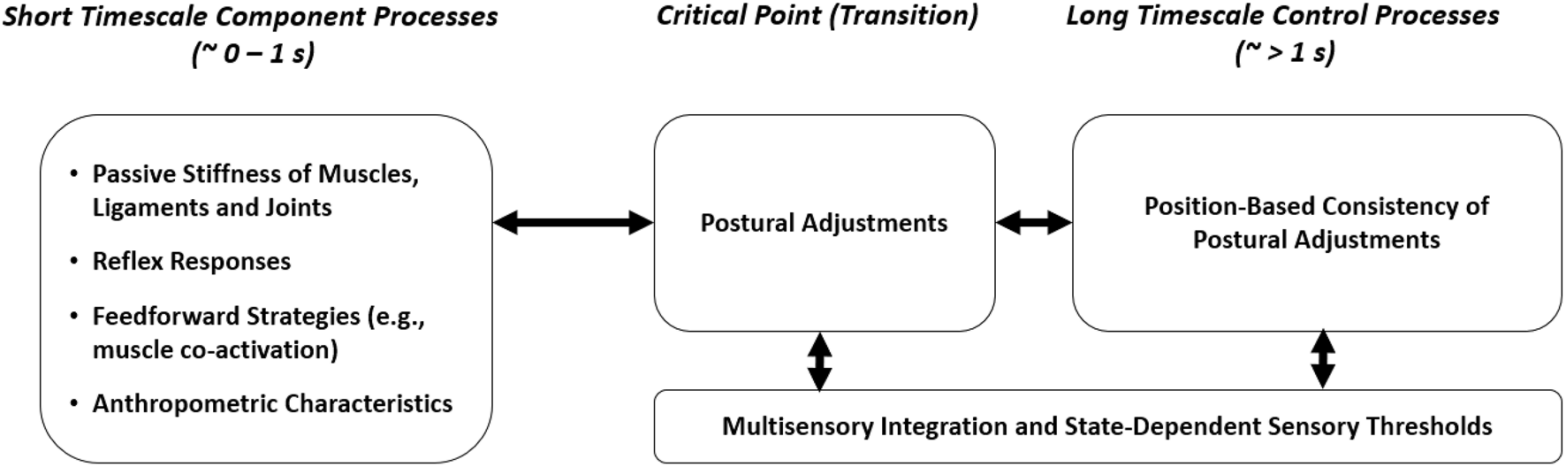
Component processes of postural control acting at different timescales.

Our findings warrant a reinterpretation of the open- and closed-loop framework proposed by Collins and De Luca^33^. Figure 4 depicts the component processes we believe to influence postural control at differential timescales. Collins & de Luca^33^ suggested that during short timescales, an open-loop control scheme is employed which leaves postural diffusion uncorrected by active mechanisms and allows for a certain level of drift. Subsequently, once a threshold is exceeded, sensory information is used to execute long-term closed-loop corrective adjustments. We argue that the open vs closed-loop distinction is important, however, control processes and their effects on SDA parameters, cannot be assigned exclusively to either one category. This interpretation echoes Peterka's^36^ claim that altered closed-loop mechanisms influence short-term diffusion in upright stance.

According to the traditional interpretation, long-term diffusion must deteriorate when sensory information is compromised. Instead, our results showed that short- but not long-term diffusion was systematically affected by the removal of visual information, with older adults even improving their long-term control under eyes closed conditions. We consider these findings clear evidence that short-term diffusion is determined by both open- and closed-loop processes. Put differently, displacement at short timescales includes trajectories where CoP drift is persistent as well as intervals where this drifting motion gets corrected and changes direction. So conceived, the critical point should not be considered the transition from open- to closed-loop mechanisms, but rather the point at which, on average, corrective feedback motion takes place. This means that the delay is not the passive result of neural processing delays but adaptively determined by the degree to which stability is jeopardized as well as the sensory information available to the postural control system. By the same token, long-term diffusion does not reflect closed-loop mechanisms in its pure form but the position-based consistency of these corrective adjustments. If there is an absolute reference point around which CoP drift occurs (an idealized stable attractor context to which stable stance comes closest), long-term diffusion will be small. In contrast, when participants must adaptively adjust their center of stability throughout time, long-term diffusion will increase considerably. Our study has several limitations. While we took a lifespan perspective our data did not cover middle adulthood. Considering that late adulthood changes in the postural control system are well under way during this period, future studies should pay more attention to this age group. Another limitation of our study is that we exclusively relied on center-of-pressure measures to assess postural control performance. This may well miss out on other important postural control descriptors like muscle activation patterns and it limits the consideration of differences in anthropometric characteristics (e.g., height, body mass) to statistical control. Future studies should aim to overcome these weaknesses by using a multi-method approach. As a final point, we specifically instructed participants to stand as still as possible during balance tasks. While this is common practice during studies, this approach may have influenced or limited the repertoire of balance strategies adopted, particularly by young adults^40^.

In conclusion, applying SDA in addition to traditional summary measures to a lifespan sample together with experimentally manipulating balance performance conditions has provided insights into underlying control mechanisms, their age-related changes, and the adaptability of the postural control system to different contexts.

## Methods

### Participants

A total of 179 children (7–14 years of age), 50 young adults (20–29 years of age), and 72 older adults (60–81 years of age) were recruited through advertisements in local stores and from the subject pool of the Max Planck Institute for Human Development in Berlin. We did not include participants who had (a) a recent fall or sports accident, (b) a history of neurological disorders or stroke (c) a neurodevelopmental disorder known to affect balance (i.e., ADHD). Two children were excluded only after testing because of orthopedic conditions leaving 299 participants for analysis. Descriptive characteristics of the final sample are presented in Table 2. The testing procedure was part of a larger series of assessments that fall out of the scope of this article, including assessments of cognitive (digit span, digit symbol substitution, HAWIE vocabulary test and letter fluency test) and sensory abilities (near/far vision, pure tone audiometry). Individual test sessions lasted between 1.5 and 2 h with multiple breaks at participants' dispositions. Participants received the equivalent of 10 Euro per hour for participation. The study was conducted in accordance with the declaration of Helsinki and it was approved by the Ethics Committee of the Max Planck Institute for Human Development. Informed consent was obtained from all participants. Informed consent was signed by parents in the case of children and adolescents.

**Table 2:** Descriptive characteristics of each age group (standard deviations).

	Children	Young adults	Older adults
N	177	50	72
age (years)	10.6 (2.1)	24.2 (2.0)	70.2 (6.2)
height (cm)	149.0 (14.6)	177.2 (10.3)	171.2 (8.4)
mass (kg)	40.6 (12.1)	70.5 (12.4)	75.7 (12.3)
bmi (kg/m^2^)	17.9 (2.6)	22.3 (2.6)	25.7 (3.5)

### Testing procedure

To measure each participant’s ability to stabilize the body’s equilibrium under different postural control demands, balance performance was assessed on a 40 × 60 cm dynamic force platform (Kistler force platform 9286AA, Kistler Instrumenten AG, Winterthur, Switzerland). The position of the body’s CoP was calculated for each millisecond (1000 Hz sampling frequency) by recording the components of the ground reaction force on the lateral, vertical, and horizontal axes. For safety reasons, handrails were adjusted at each side of the platform at waist level and participants were secured by safety belts known from mountain climbing. Participants were instructed to stand as still as possible while gazing at a fixation cross for 30 s. Similar to the Romberg’s test, arms were positioned forward and parallel with the palms facing upwards^41^. During stable stance and eyes closed conditions, feet were positioned parallel 10 cm apart. During tandem stance, feet were positioned heel-to-toe. To make sure that participants understood the task, visual feedback was provided after each trial. The assessment of balance performance started with one warm-up trial with real-time visual feedback, followed by three trials in stable stance and one trial in eyes closed and tandem stance conditions.

### Pre-processing

**Figure 5:**
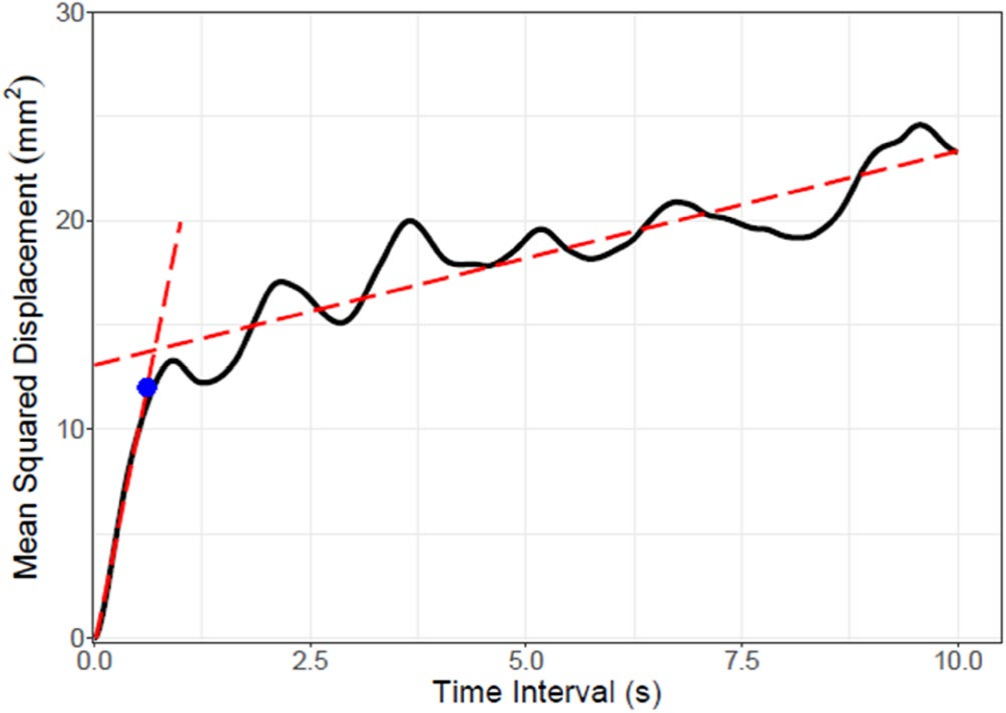
Graphical example of stabilogram diffusion plot in a representative subject in stable stance. Red dotted lines represent the slopes of the short and long timescale regions. The critical point is depicted in blue and represents the separation between short and long timescale regions.

All preprocessing and statistics were performed using R 4.1.2 (R Core Team, 2021). A fourth order low-pass Butterworth filter of 13 Hz was applied to the CoP data. Planar path length represented the total planar CoP displacement during each trial. The ellipse area was calculated by using the princomp function in R to determine the major and minor axes of the ellipse. Following Oliveira et al., the axes lengths of the ellipse are then defined as 1.96 standard deviations in the respective directions, thus including 95% of the datapoints along each axis^42^. Evaluation of postural control also included the stabilogram diffusion analysis (SDA) as pioneered by Collins and De Luca^33^ (Fig. 5). For this method, we calculated the mean squared displacement of all pairs of data points separated by time intervals ranging from 1 to 10,000 ms. Subsequently, we determined the critical point using the Segmented function from the Segmented package^43^ in R to calculate the breakpoint of a piecewise linear regression fit onto the data. The x and y values of the breakpoint respectively representing the critical time intervals and the critical mean squared displacement. All time intervals up to the critical point are considered short timescales whereas the others are considered long timescales. In order to increase reliability of the piecewise regression method, time intervals from 50 to 3000 ms were used to determine the critical point. The slopes of the linear regressions fit on short and long timescale regions were used to quantify the short and long-term diffusion coefficients respectively.

### Statistics

We selected linear mixed-effect models (LMMs) to analyze the data. LMMs allow for multiple grouping hierarchies (main effects as well as nested within-group effects) while also taking advantage of multiple correlating measures assessed within the same subject (three experimental conditions)^44^. Separate LMMs were fit for planar path length, ellipse area and the four SDA parameters using the lmer^45^ and lmerTest^46^ packages. Inspection of distributions of dependent variables (see Supplementary Fig. 1–6) using the Box-Cox method^47^ in R advised logarithmic transformations or, for the long-term diffusion coefficient, a negative power transformation (λ = − 0.2, ɣ = 0.8). We excluded 36 trials (< 3%) from data analysis due to deficient fitting or technical issues. For stable stance, the mean of each of the variables was calculated and used for the following analyses.

We followed the parsimonious model selection procedure proposed by Bates et al.^45^. The initial LMM included fixed effects for condition (stable stance, eyes closed, tandem stance), age group (children, young adults, older adults), their interactions and subject (intercept) as a random factor. Within-age-group differences related to sex (female vs male), age (centered), their interaction and norm-referenced BMI^48,49^ were included as nested factors. We specified two a priori contrasts comparing stable stance as reference condition with eyes-closed and tandem stance conditions, respectively. To analyze age-related differences we first contrasted young adults with children on the one hand and older adults on the other (see Supplementary Table S1-S6). In a second step, we contrasted children to young and older adults in a separate set of LMMs (see Supplementary Tables S7–S12) in which we adjusted the alpha level for multiple testing (i.e., 0.025). Post-hoc tests included Bonferroni adjusted t-tests. We optimized models based on LMM's goodness of fit using the likelihood ratio test. We removed fixed effects without reliable contributions and we expanded the random effect structure by allowing subject by condition interactions. Results regarding model selection and nested effects of sex and norm-referenced BMI are addressed in the Supplementary Notes.

## Supplementary Information

Supplementary Information.

## Data Availability

The datasets generated and analyzed during the current study are available from the corresponding author on reasonable request.
