# Supplementary material for: Lifespan changes in postural control
Source: Sci Rep. 2023 Jan 11;13:541. doi: 10.1038/s41598-022-26934-0 (PMC9834247; doi:10.1038/s41598-022-26934-0)
Supplement: Supplementary file 1 — Supplementary Information. [file 41598_2022_26934_MOESM1_ESM.pdf]

Supplementary Notes

Model Selection

Nested effects of age improved the model fit in all parameters ($\chi 2(3) \geq 16.933$, $p<0.001$) except from the critical time interval ($\chi 2(3) = 5.386$, $p=0.146$). Sex contributed to the goodness of fit in the planar path lengths, ellipse area, short-term diffusion coefficient and critical displacement ($\chi 2(3) \geq 11.032$, $p \geq 0.012$). BMI only contributed to the model of the long-term diffusion coefficient ($\chi 2(3)= 7.8233$, $p=0.050$). Lastly, expanding the random effect structure by allowing subject by condition interaction improved the goodness of fit significantly in all parameters ($\chi 2(2) \geq 17.146$, $p<0.001$) except from the critical time interval ($\chi 2(2) = 0.6917$, $p=0.708$).

Nested effects of Sex

A nested effect of sex revealed that, in children ($z=4.063$, $p<0.001$) and younger adults ($z=2.067$, $p= 0.040$), female participants showed less planar displacement when compared with male participants. The latter effect was also observed for children in the ellipse area; female participants showed smaller areas of sway when compared with male participants ($z=3.212$, $p= 0.001$). Nested effects of sex reflected higher short-term diffusion ($z=3.985$, $p<0.001$) and larger critical displacement ($z=4.034$, $p<0.001$) in boys than in girls.

Effects	Estimate	Std. Error	z	p
Fixed effects				
Eyes closed - stable stance	0,22***	0,006	35,294	<.001
Tandem - stable stance	0,493***	0,008	62,898	<.001
Children - young adults	0,125***	0,018	6,767	<.001
Older adults - young adults	0,145***	0,021	6,872	<.001
Interactions				
Eyes closed - stable stance x children - young adults	-0,025	0,015	-1,693	0,091
Tandem - stable stance x children - young adults	-0,028	0,019	-1,488	0,138
Eyes closed - stable stance x older adults - young adults	0,076***	0,017	4,373	<.001
Tandem - stable stance x older adults - young adults	0,158***	0,022	7,279	<.001
Nested Effects Children				
Age	-0,013**	0,004	-3,235	<.01
Sex	-0,07***	0,017	-4,063	<.001
Nested Effects Young Adults				
Age	-0,009	0,008	-1,086	0,279
Sex	-0,068*	0,033	-2,067	<.05
Nested Effects Older Adults				
Age	0,015***	0,002	6,797	<.001
Sex	-0,027	0,027	-0,996	0,320

Supplementary Table 1. Effect estimates for the final linear-mixed model using planar path length as a dependent variable (*p < .05. **p < .001).

Effects	Estimate	Std. Error	z	p
Fixed effects				
Eyes closed - stable stance	0,274***	0,016	17,467	<.001
Tandem - stable stance	0,562***	0,018	31,027	<.001
Children - young adults	0,317***	0,035	9,118	<.001
Older adults - young adults	0,304***	0,040	7,588	<.001
Interactions				
Eyes closed - stable stance x children - young adults	-0,057	0,038	-1,496	0,136
Tandem - stable stance x children - young adults	-0,021	0,044	-0,473	0,637
Eyes closed - stable stance x older adults - young adults	-0,001	0,044	-0,017	0,986
Tandem - stable stance x older adults - young adults	0,15**	0,050	2,991	<.01
Nested Effects Children				
Age	-0,034***	0,008	-4,450	<.001
Sex	-0,105**	0,033	-3,212	<.01
Nested Effects Young Adults				
Age	-0,019	0,016	-1,230	0,220
Sex	-0,093	0,062	-1,501	0,134
Nested Effects Older Adults				
Age	0,024***	0,004	5,862	<.001
Sex	-0,016	0,051	-0,307	0,759

Supplementary Table 2. Effect estimates for the final linear-mixed model using the ellipse area as a dependent variable

(*p < .05. **p < .001).

Effects	Estimate	Std. Error	z	p
Fixed effects				
Eyes closed - stable stance	0,43***	0,016	27,687	<.001
Tandem - stable stance	0,862***	0,017	49,598	<.001
Children - young adults	0,222***	0,034	6,488	<.001
Older adults - young adults	0,283***	0,039	7,197	<.001
Interactions				
Eyes closed - stable stance x children - young adults	-0,053	0,037	-1,403	0,162
Tandem - stable stance x children - young adults	-0,022	0,042	-0,518	0,605
Eyes closed - stable stance x older adults - young adults	0,097*	0,043	2,240	<.05
Tandem - stable stance x older adults - young adults	0,238***	0,048	4,939	<.001
Nested Effects Children				
Age	-0,032***	0,008	-4,146	<.001
Sex	-0,128***	0,032	-3,985	<.001
Nested Effects Young Adults				
Age	-0,014	0,016	-0,911	0,363
Sex	-0,077	0,061	-1,268	0,206
Nested Effects Older Adults				
Age	0,029***	0,004	7,187	<.001
Sex	-0,023	0,050	-0,458	0,647

Supplementary Table 3. Effect estimates for the final linear-mixed model using the short-term diffusion coefficient as a dependent variable (*p < .05. **p < .01 ***p < .001).

Effects	Estimate	Std. Error	z	p
Fixed effects				
Eyes closed - stable stance	0,021	0,054	0,383	0,702
Tandem - stable stance	0,377***	0,055	6,875	<.001
Children - young adults	0,468***	0,077	6,057	<.001
Older adults - young adults	0,298***	0,089	3,352	<.001
Interactions				
Eyes closed - stable stance x children - young adults	-0,03	0,131	-0,229	0,819
Tandem - stable stance x children - young adults	-0,032	0,132	-0,241	0,810
Eyes closed - stable stance x older adults - young adults	-0,32*	0,151	-2,113	<.05
Tandem - stable stance x older adults - young adults	0,184	0,152	1,207	0,228
Nested Effects Children				
Age	-0,05**	0,017	-2,916	<.01
Bmi	0,071	0,043	1,656	0,099
Nested Effects Young Adults				
Age	-0,025	0,035	-0,717	0,474
Bmi	-0,106	0,088	-1,212	0,227
Nested Effects Older Adults				
Age	0,027**	0,009	2,903	<.01
Bmi	0,132	0,068	1,943	0,053

Supplementary Table 4. Effect estimates for the final linear-mixed model using the long-term diffusion coefficient as a dependent variable (*p < .05. **p < .01 ***p < .001).

Effects	Estimate	Std. Error	z	p
Fixed effects				
Eyes closed - stable stance	0,073***	0,013	5,424	<.001
Tandem - stable stance	-0,218***	0,013	-16,146	<.001
Children - young adults	0,031	0,019	1,679	0,094
Older adults - young adults	0,023	0,021	1,094	0,275
Interactions				
Eyes closed - stable stance x children - young adults	-0,09**	0,033	-2,756	<.01
Tandem - stable stance x children - young adults	-0,027	0,033	-0,830	0,407
Eyes closed - stable stance x older adults - young adults	-0,064	0,037	-1,702	0,089
Tandem - stable stance x older adults - young adults	-0,146***	0,037	-3,904	<.001

Supplementary Table 5. Effect estimates for the final linear-mixed model using critical time interval as a dependent variable (*p < .05. **p < .01 ***p < .001).

Effects	Estimate	Std. Error	z	p
Fixed effects				
Eyes closed - stable stance	0,542***	0,014	37,516	<.001
Tandem - stable stance	0,677***	0,015	43,776	<.001
Children - young adults	0,27***	0,033	8,196	<.001
Older adults - young adults	0,317***	0,038	8,368	<.001
Interactions				
Eyes closed - stable stance x children - young adults	-0,163***	0,035	-4,680	<.001
Tandem - stable stance x children - young adults	-0,057	0,037	-1,527	0,128
Eyes closed - stable stance x older adults - young adults	0,019	0,040	0,476	0,634
Tandem - stable stance x older adults - young adults	0,085*	0,043	1,987	<.05
Nested Effects Children				
Age	-0,036***	0,007	-4,948	<.001
Sex	-0,125***	0,031	-4,034	<.001
Nested Effects Young Adults				
Age	-0,007	0,015	-0,449	0,654
Sex	-0,069	0,059	-1,186	0,236
Nested Effects Older Adults				
Age	0,024***	0,004	6,191	<.001
Sex	-0,006	0,049	-0,128	0,898

Supplementary Table 6. Effect estimates for the final linear-mixed model using critical displacement as a dependent variable (*p < .05. **p < .01 ***p < .001).

Effects	Estimate	Std. Error	z	p
Fixed effects				
Eyes closed vs stable stance	0,22***	0,006	35,294	<.001
Tandem vs stable stance	0,493***	0,008	62,898	<.001
Children vs young adults	0,125***	0,018	6,767	<.001
Children vs older adults	-0,021	0,016	1,298	0,195
Interactions				
Eyes closed vs stable stance x children vs young adults	-0,025	0,015	-1,693	0,091
Tandem vs stable stance x children vs young adults	-0,028	0,019	-1,488	0,138
Eyes closed vs stable stance x children vs older adults	-0,101***	0,013	7,680	<.001
Tandem vs stable stance x children vs older adults	-0,186***	0,017	11,238	<.001
Nested Effects Children				
Age	-0,013**	0,004	-3,235	<.01
Sex	-0,07***	0,017	-4,063	<.001
Nested Effects Young Adults				
Age	-0,009	0,008	-1,086	0,279
Sex	-0,068*	0,033	-2,067	<.05
Nested Effects Older Adults				
Age	0,015***	0,002	6,797	<.001
Sex	-0,027	0,027	-0,996	0,320

Supplementary Table 7. Effect estimates for the post-hoc linear-mixed model using planar pathlengths as a dependent variable (*p < .05. **p < .001).

Effects	Estimate	Std. Error	z	p
Fixed effects				
Eyes closed vs stable stance	0,274***	0,016	17,467	<.001
Tandem vs stable stance	0,562***	0,018	31,027	<.001
Children vs young adults	0,317***	0,035	9,118	<.001
Children vs older adults	0,014	0,030	-0,452	0,652
Interactions				
Eyes closed vs stable stance x children vs young adults	0,057	0,038	1,496	0,136
Tandem vs stable stance x children vs young adults	0,021	0,044	0,473	0,637
Eyes closed vs stable stance x children vs older adults	-0,056	0,033	1,684	0,093
Tandem vs stable stance x children vs older adults	-0,171***	0,038	4,461	<.001
Nested Effects Children				
Age	-0,034***	0,008	-4,450	<.001
Sex	-0,105**	0,033	-3,212	<.01
Nested Effects Young Adults				
Age	-0,019	0,016	-1,230	0,220
Sex	-0,093	0,062	-1,501	0,134
Nested Effects Older Adults				
Age	0,024***	0,004	5,862	<.001
Sex	-0,016	0,051	-0,307	0,759

Supplementary Table 8. Effect estimates for the post-hoc linear-mixed model using the ellipse area as a dependent variable (*p < .05. **p < .001).

Effects	Estimate	Std. Error	z	p
Fixed effects				
Eyes closed vs stable stance	0,43***	0,016	27,687	<.001
Tandem vs stable stance	0,862***	0,017	49,598	<.001
Children vs young adults	0,222***	0,034	-6,488	<.001
Children vs older adults	-0,061*	0,030	2,045	0.042
Interactions				
Eyes closed vs stable stance x children vs young adults	-0,053	0,037	1,403	0,162
Tandem vs stable stance x children vs young adults	-0,022	0,042	0,518	0,605
Eyes closed vs stable stance x children vs older adults	-0,149***	0,033	4,544	<.001
Tandem vs stable stance x children vs older adults	-0,26***	0,037	7,065	<.001
Nested Effects Children				
Age	-0,032***	0,008	-4,146	<.001
Sex	-0,128***	0,032	-3,985	<.001
Nested Effects Young Adults				
Age	-0,014	0,016	-0,911	0,363
Sex	-0,077	0,061	-1,268	0,206
Nested Effects Older Adults				
Age	0,029***	0,004	7,187	<.001
Sex	-0,023	0,050	-0,458	0,647

Supplementary Table 9. Effect estimates for the post-hoc linear-mixed model using the short-term diffusion coefficient as a dependent variable (*p < .05. **p < .01 ***p < .001).

Effects	Estimate	Std. Error	z	p
Fixed effects				
Eyes closed vs stable stance	0,021	0,054	0,383	0,702
Tandem vs stable stance	0,377***	0,055	6,875	<.001
Children vs young adults	0,468***	0,077	-6,057	<.001
Children vs older adults	0,17*	0,068	-2,517	0.012
Interactions				
Eyes closed vs stable stance x children vs young adults	-0,03	0,131	0,229	0,819
Tandem vs stable stance x children vs young adults	-0,032	0,132	0,241	0,810
Eyes closed vs stable stance x children vs older adults	0,29*	0,115	-2,517	0.012
Tandem vs stable stance x children vs older adults	-0,215	0,116	1,859	0,064
Nested Effects Children				
Age	-0,05**	0,017	-2,916	<.01
Bmi	0,071	0,043	1,656	0,099
Nested Effects Young Adults				
Age	-0,025	0,035	-0,717	0,474
Bmi	-0,106	0,088	-1,212	0,227
Nested Effects Older Adults				
Age	0,027**	0,009	2,903	<.01
Bmi	0,132	0,068	1,943	0,053

Supplementary Table 10. Effect estimates for the post-hoc linear-mixed model using the long-term diffusion coefficient as a dependent variable (*p < .05. **p < .01 ***p < .001).

Effects	Estimate	Std. Error	z	p
Fixed effects				
Eyes closed vs stable stance	0,073***	0,013	5,424	<.001
Tandem vs stable stance	-0,218***	0,013	-16,146	<.001
Children vs young adults	-0,031	0,019	1,679	0,094
Children vs older adults	0,008	0,016	-0,481	0,631
Interactions				
Eyes closed vs stable stance x children vs young adults	-0,09**	0,033	-2,756	<.01
Tandem vs stable stance x children vs young adults	-0,027	0,033	-0,830	0,407
Eyes closed vs stable stance x children vs older adults	-0,026	0,028	0,909	0,364
Tandem vs stable stance x children vs older adults	0,119***	0,029	-4,176	<.001

Supplementary Table 11. Effect estimates for the post-hoc linear-mixed model using critical time interval as a dependent variable (*p < .05. **p < .01 ***p < .001).

**Effects**	**Estimate**	**Std. Error**	**z**	**p**
Fixed effects				
Eyes closed vs stable stance	0,542***	0,014	37,516	<.001
Tandem vs stable stance	0,677***	0,015	43,776	<.001
Children vs young adults	0,27***	0,033	8,196	<.001
Children vs older adults	-0,047	0,029	1,630	0,104
Interactions				
Eyes closed vs stable stance x children vs young adults	-0,163***	0,035	-4,680	<.001
Tandem vs stable stance x children vs young adults	-0,057	0,037	-1,527	0,128
Eyes closed vs stable stance x children vs older adults	-0,182***	0,031	5,965	<.001
Tandem vs stable stance x children vs older adults	-0,142***	0,033	4,346	<.001
Nested Effects Children				
Age	-0,036***	0,007	-4,948	<.001
Sex	-0,125***	0,031	-4,034	<.001
Nested Effects Young Adults				
Age	-0,007	0,015	-0,449	0,654
Sex	-0,069	0,059	-1,186	0,236
Nested Effects Older Adults				
Age	0,024***	0,004	6,191	<.001
Sex	-0,006	0,049	-0,128	0,898

Supplementary Table 12. Effect estimates for the post-hoc linear-mixed model using critical displacement as a dependent variable (*p < .05. **p < .01 ***p < .001).

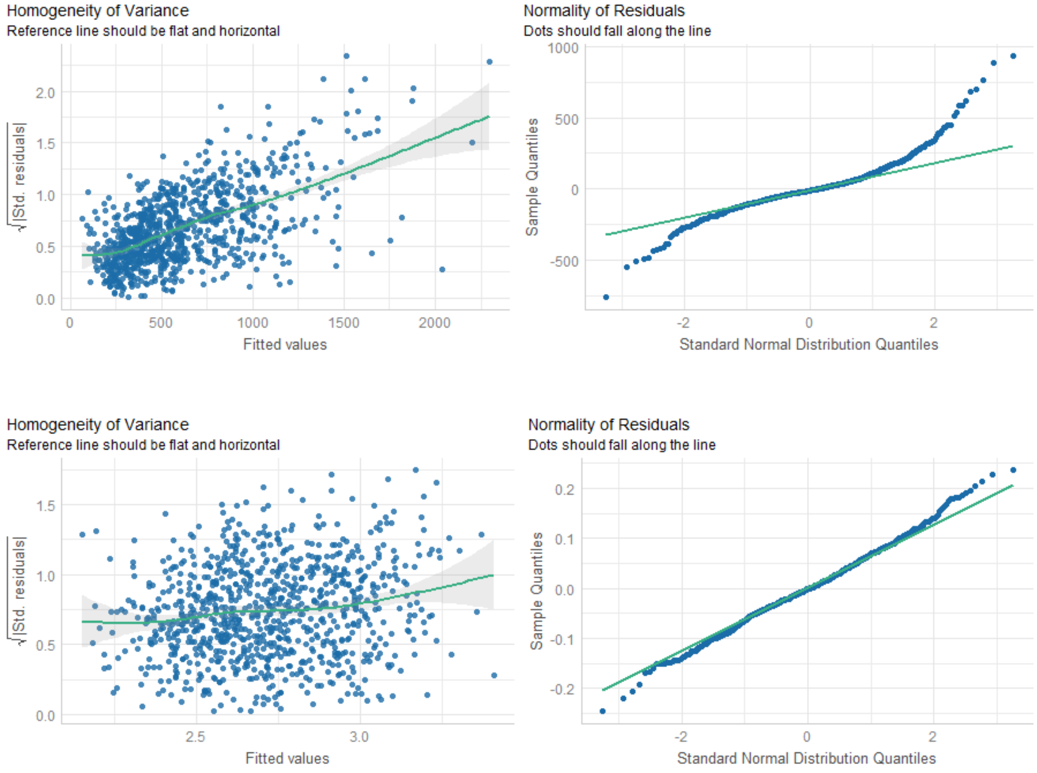

Supplementary Figure 1. Visualization of homogeneity of variance and normality of residuals of the planar pathlengths before (upper panels) and after (lower panels) transformation.

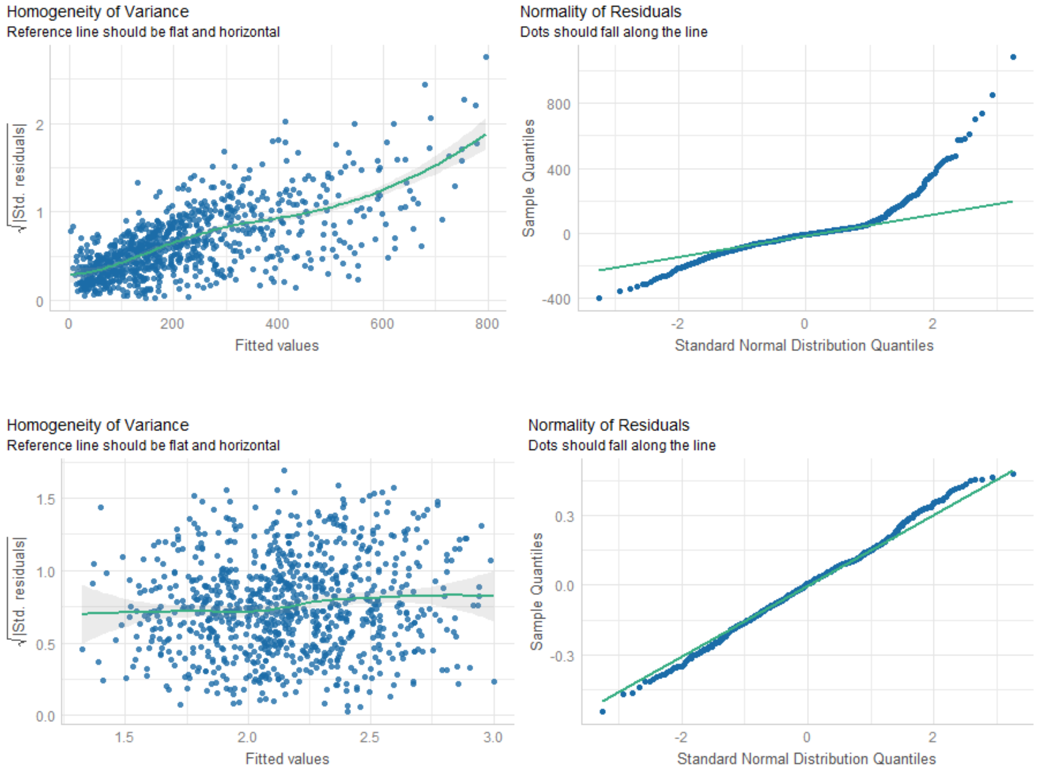

Supplementary Figure 2. Visualization of homogeneity of variance and normality of residuals of the ellipse area before (upper panels) and after (lower panels) transformation.

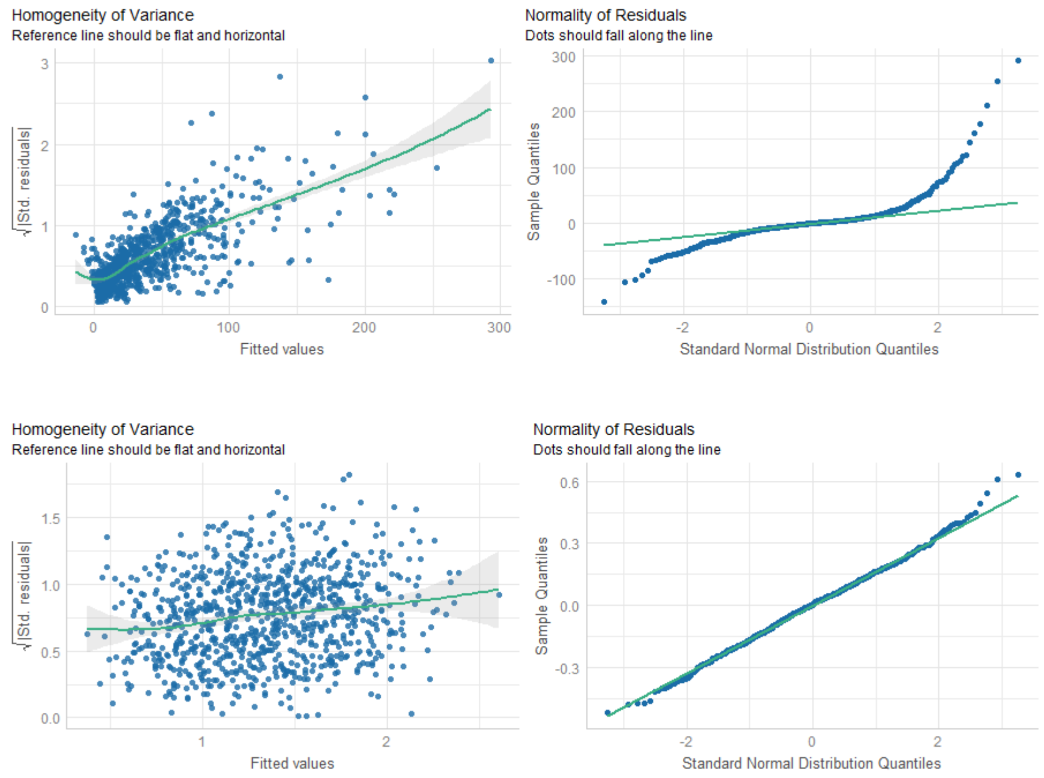

Supplementary Figure 3. Visualization of homogeneity of variance and normality of residuals of the short-term diffusion coefficient before (upper panels) and after (lower panels) transformation.

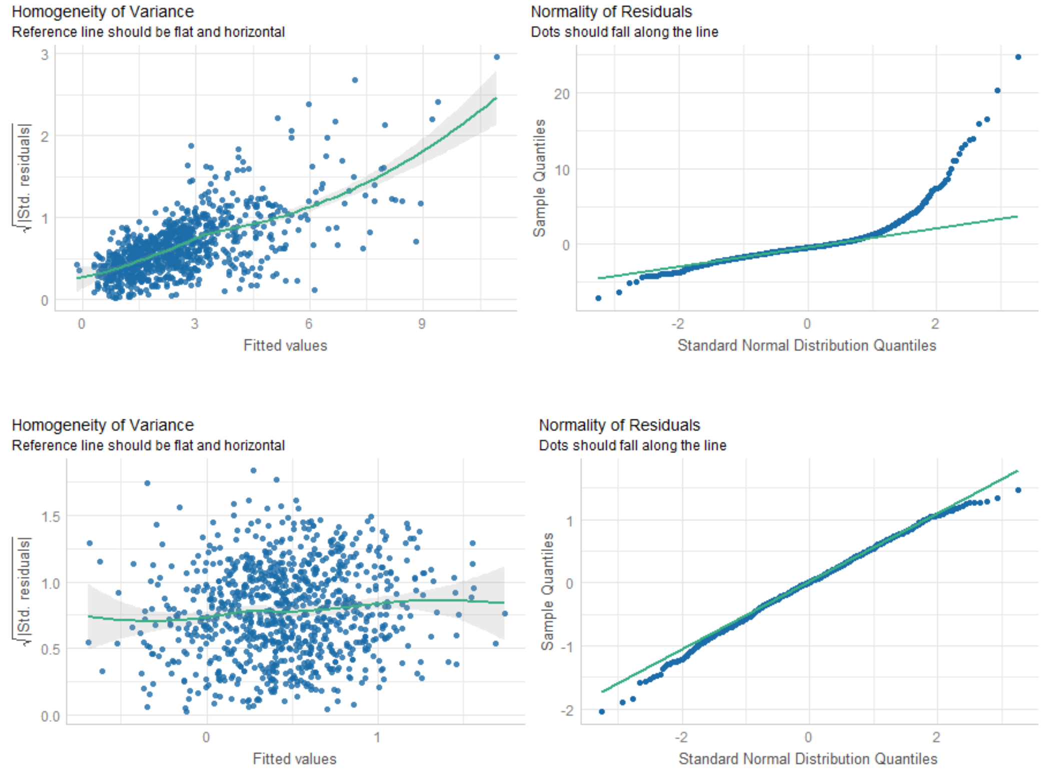

Supplementary Figure 4. Visualization of homogeneity of variance and normality of residuals of the long-term diffusion coefficient before (upper panels) and after (lower panels) transformation.

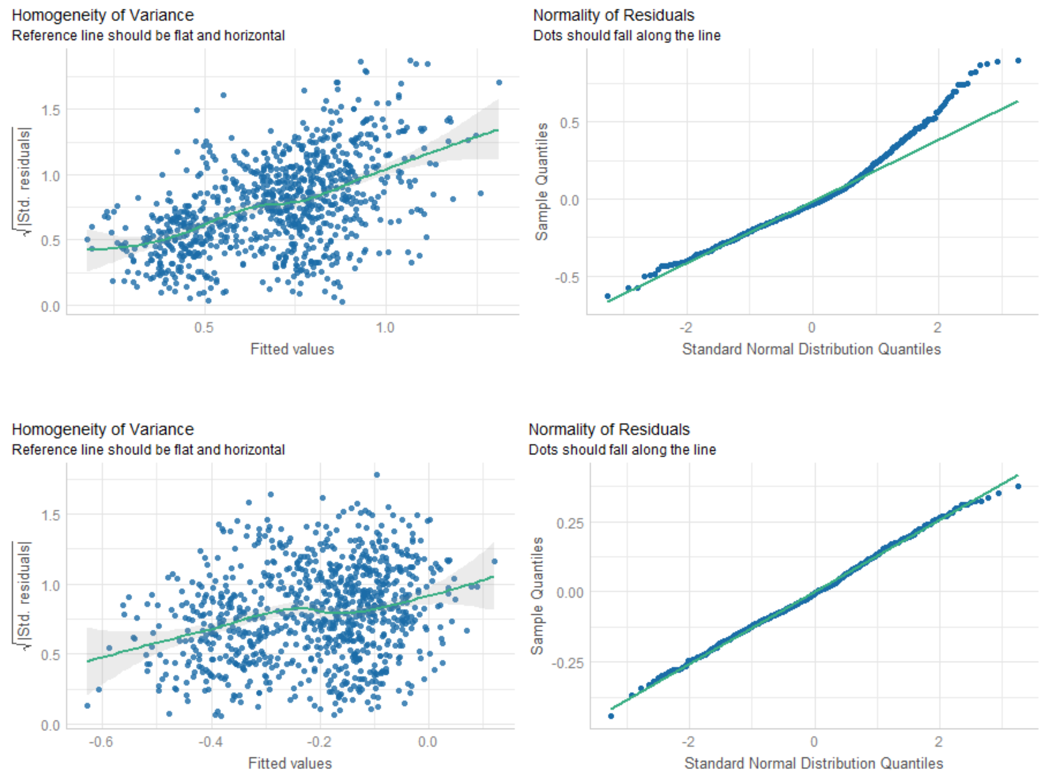

Supplementary Figure 5. Visualization of homogeneity of variance and normality of residuals of the critical time interval before (upper panels) and after (lower panels) transformation.

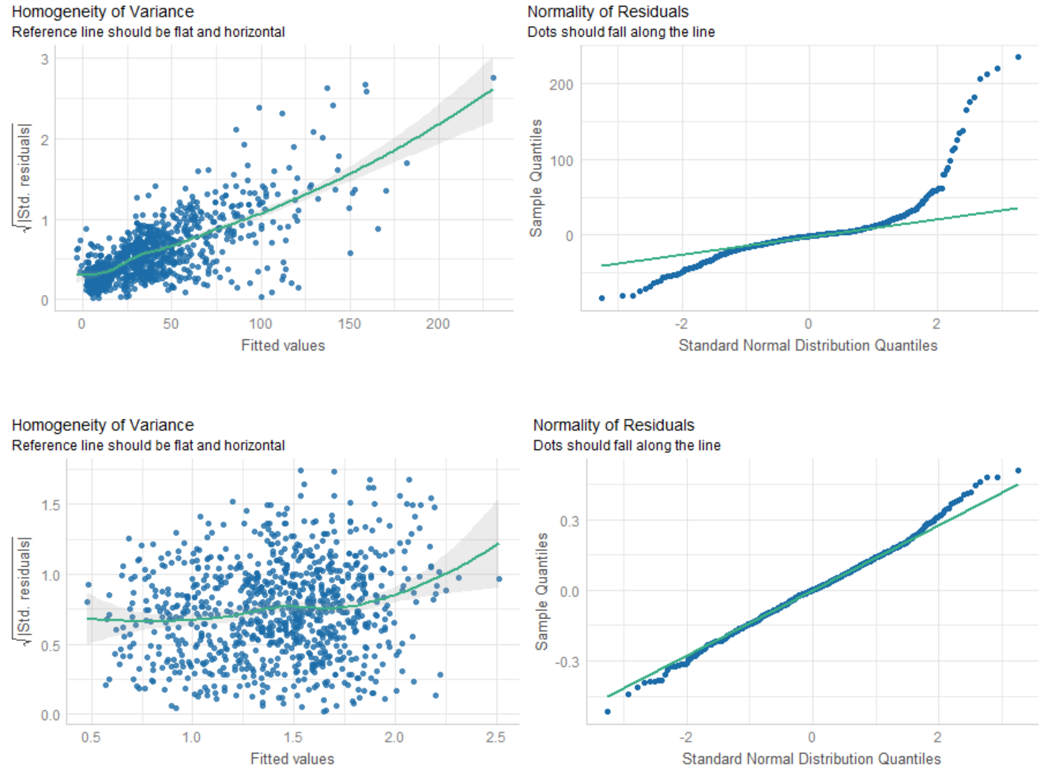

Supplementary Figure 6. Visualization of homogeneity of variance and normality of residuals of the critical mean squared displacement before (upper panels) and after (lower panels) transformation.
